# 2,5-Dibromo­indan-1-ol

**DOI:** 10.1107/S1600536812035829

**Published:** 2012-08-25

**Authors:** Ísmail Çelik, Mehmet Akkurt, Makbule Yilmaz, Ahmet Tutar, Ramazan Erenler, Santiago García-Granda

**Affiliations:** a Department of Physics, Faculty of Sciences, Cumhuriyet University, 58140 Sivas, Turkey; b Department of Physics, Faculty of Sciences, Erciyes University, 39039 Kayseri, Turkey; c Duzce University, Faculty of Art and Science, Department of Chemistry, TR-81620 Duzce, Turkey; d Sakarya University, Faculty of Art and Science, Department of Chemistry, TR-54187 Adapazarı, Turkey; e Gaziosmanpasa University, Faculty of Art and Science, Department of Chemistry, TR-60240 Tokat, Turkey; f Departamento Química Física y Analítica, Facultad de Química, Universidad Oviedo, C/ Julián Clavería, 8, 33006 Oviedo (Asturias), Spain

## Abstract

In the title compound, C_9_H_8_Br_2_O, the cyclo­pentene ring adopts an envelope conformation with the brominated C atom as the flap. In the crystal, mol­ecules are linked by strong O—H⋯O hydrogen bonds into zigzag *C*(4) chains along [010]. In addition, a C—H⋯π inter­action involving the benzene ring and the H atom attached to the hy­droxy­lated C atom is observed.

## Related literature

For bromination of hydro­carbons, see: Cakmak *et al.* (2006 ▶); Erenler & Cakmak (2004 ▶); Erenler *et al.* (2006 ▶). For the pharmacological and medicinal proparties of indanes, see: Mitrochkine *et al.* (1995 ▶); Catto *et al.* (2010 ▶); Wu (2006 ▶); McClure *et al.* (2011 ▶) and for their use in natural product chemistry, see: Snyder & Brill (2011 ▶). For a similar structure, see: Çelik *et al.* (2012 ▶). For puckering parameters, see: Cremer & Pople (1975 ▶). For hydrogen-bond motifs, see: Bernstein *et al.* (1995 ▶).
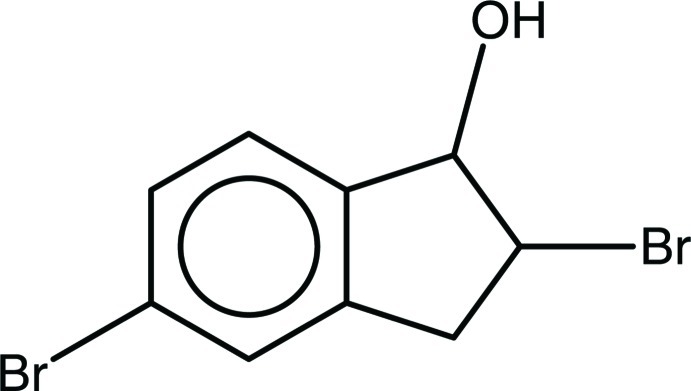


## Experimental

### 

#### Crystal data

C_9_H_8_Br_2_O
*M* *_r_* = 291.95
Monoclinic, 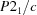

*a* = 9.5137 (10) Å
*b* = 4.8991 (7) Å
*c* = 20.249 (3) Å
β = 94.165 (10)°
*V* = 941.3 (2) Å^3^
*Z* = 4
Cu *K*α radiation
μ = 10.50 mm^−1^
*T* = 299 K
0.17 × 0.01 × 0.01 mm

#### Data collection

Agilent Xcalibur Ruby Gemini diffractometer
Absorption correction: refined from Δ*F* (*XABS2*; Parkin *et al.*, 1995 ▶)*T*_min_ = 0.882, *T*_max_ = 0.900
1777 measured reflections
1777 independent reflections
733 reflections with *I* > 2σ(*I*)

#### Refinement

*R*[*F* ^2^ > 2σ(*F* ^2^)] = 0.082
*wR*(*F* ^2^) = 0.228
*S* = 1.00
1777 reflections
79 parameters
H-atom parameters constrained
Δρ_max_ = 0.68 e Å^−3^
Δρ_min_ = −0.78 e Å^−3^

### 

Data collection: *CrysAlis PRO* (Agilent, 2011 ▶); cell refinement: *CrysAlis PRO*; data reduction: *CrysAlis PRO*; program(s) used to solve structure: *SIR97* (Altomare *et al.*, 1999 ▶); program(s) used to refine structure: *SHELXL97* (Sheldrick, 2008 ▶); molecular graphics: *ORTEP-3 for Windows* (Farrugia, 1999 ▶); software used to prepare material for publication: *WinGX* (Farrugia, 1997 ▶) and *PLATON* (Spek, 2009 ▶).

## Supplementary Material

Crystal structure: contains datablock(s) global, I. DOI: 10.1107/S1600536812035829/pk2441sup1.cif

Structure factors: contains datablock(s) I. DOI: 10.1107/S1600536812035829/pk2441Isup2.hkl

Supplementary material file. DOI: 10.1107/S1600536812035829/pk2441Isup3.cml

Additional supplementary materials:  crystallographic information; 3D view; checkCIF report

## Figures and Tables

**Table 1:** Hydrogen-bond geometry (Å, °) *Cg*2 is the centroid of the C1–C6 benzene ring.

*D*—H⋯*A*	*D*—H	H⋯*A*	*D*⋯*A*	*D*—H⋯*A*
O1—H1⋯O1^i^	0.82	1.91	2.713 (14)	165
C9—H9⋯*Cg*2^ii^	0.98	2.67	3.629 (16)	166

Symmetry codes: (i) 
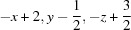
; (ii) 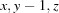.

**Fig. 1.**
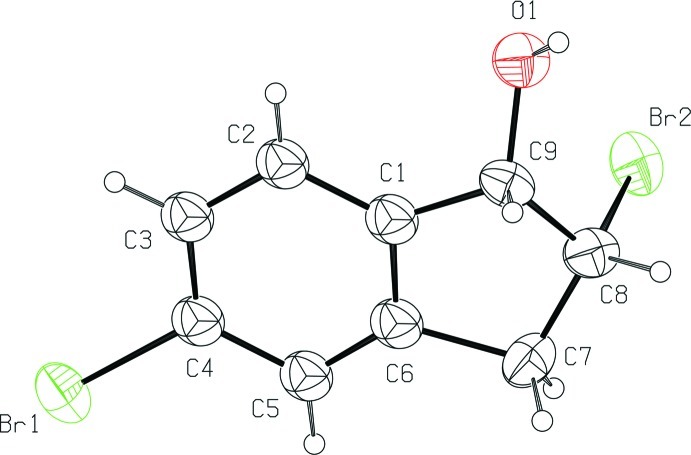
An ORTEP plot of (I) with the atom numbering scheme. Displacement ellipsoids for non-H atoms are drawn at the 30% probability level.

**Fig. 2.**
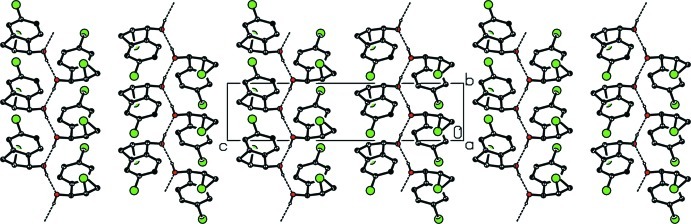
View of the packing and hydrogen bonding of (I), along the a axis. H atoms not involved in hydrogen bonding are omitted for the sake of clarity.

## supplementary crystallographic information

### Comment

Bromination of hydrocarbons are important processes in synthetic chemistry (Cakmak *et al.*, 2006; Erenler *et al.*, 2006; Erenler & Cakmak, 2004). Indanes are an important class of molecules due to their pharmacological and medicinal proparties (Mitrochkine *et al.*, 1995; Catto *et al.*, 2010; Wu, 2006; McClure *et al.*, 2011) as well as in natural product chemistry (Snyder & Brill, 2011).

In the title compound (I), (Fig. 1), the five-membered C1/C6–C9 cyclopentene ring exhibits an envelope conformation with C8 at the tip of the envelope [the puckering parameters (Cremer & Pople, 1975) are Q(2) = 0.289 (17) Å and φ(2) = 290 (3) °]. All bond lengths and bond angles in (I) are in the normal range and are in good agreement with those reported in a similar structure (Çelik *et al.*, 2012).

In the crystal, pairs of strong O—H···O hydrogen bonds connect the molecules, forming zigzag *C*(4) chains propagating along the *b* axis (Bernstein *et al.*, 1995; Table 1, Fig. 2). In addition, a C—H···π interaction with the benzene ring is also found (Table 1).

### Experimental

To a stirred solution of tribromide (1.0 g, 2.8 mmol) in THF (10 ml) was added a solution of AgClO_4_.H_2_O (0.82 g, 3.64 mmol) in aqueous THF (5 ml THF / 2 ml H_2_O). The resulting mixture was stirred at room temperature for 6 h. The precipitated AgBr was removed by filtration and then the solution was dried over calcium chloride. After removal of the solvent, the residue was purified by silica gel column chromatography. Elution with hexane/ethyl acetate (4:1) afforded the 2,5-dibromo-1-hdyroxylindane (0.59 g, 72%).

### Refinement

H-atoms were positioned geometrically and refined using a riding model with O—H = 0.82 Å, C—H = 0.93–0.98 Å, and with *U*_iso_(H) = 1.2 or 1.5*U*_eq_(C,*O*).

### Crystal data

**Table:**

C_9_H_8_Br_2_O	*F*(000) = 560
*M**_r_* = 291.95	*D*_x_ = 2.060 Mg m^−^^3^
Monoclinic, *P*2_1_/*c*	Cu *K*α radiation, λ = 1.5418 Å
Hall symbol: -P 2ybc	Cell parameters from 378 reflections
*a* = 9.5137 (10) Å	θ = 4.4–70.4°
*b* = 4.8991 (7) Å	µ = 10.50 mm^−^^1^
*c* = 20.249 (3) Å	*T* = 299 K
β = 94.165 (10)°	Prism, colourless
*V* = 941.3 (2) Å^3^	0.17 × 0.01 × 0.01 mm
*Z* = 4	

### Data collection

**Table:**

Agilent Xcalibur Ruby Gemini diffractometer	1777 independent reflections
Radiation source: Enhance (Cu) X-ray Source	733 reflections with *I* > 2σ(*I*)
Graphite monochromator	*R*_int_ = 0.000
Detector resolution: 10.2673 pixels mm^-1^	θ_max_ = 70.6°, θ_min_ = 4.4°
ω scans	*h* = −11→11
Absorption correction: part of the refinement model (Δ*F*) (*XABS2*; Parkin *et al.*, 1995)	*k* = 0→5
*T*_min_ = 0.882, *T*_max_ = 0.900	*l* = 0→24
1777 measured reflections	

### Refinement

**Table:**

Refinement on *F*^2^	Primary atom site location: structure-invariant direct methods
Least-squares matrix: full	Secondary atom site location: difference Fourier map
*R*[*F*^2^ > 2σ(*F*^2^)] = 0.082	Hydrogen site location: inferred from neighbouring sites
*wR*(*F*^2^) = 0.228	H-atom parameters constrained
*S* = 1.00	*w* = 1/[σ^2^(*F*_o_^2^) + (0.0341*P*)^2^] where *P* = (*F*_o_^2^ + 2*F*_c_^2^)/3
1777 reflections	(Δ/σ)_max_ < 0.001
79 parameters	Δρ_max_ = 0.68 e Å^−^^3^
0 restraints	Δρ_min_ = −0.78 e Å^−^^3^

### Special details

**Table:**

Experimental. Absorption correction: XABS2 (Parkin *et al.*, 1995); Quadratic fit to sin(theta)/lambda - 18 parameters
Geometry. Bond distances, angles *etc*. have been calculated using the rounded fractional coordinates. All su's are estimated from the variances of the (full) variance-covariance matrix. The cell e.s.d.'s are taken into account in the estimation of distances, angles and torsion angles
Refinement. Refinement on *F*^2^ for ALL reflections except those flagged by the user for potential systematic errors. Weighted *R*-factors *wR* and all goodnesses of fit *S* are based on *F*^2^, conventional *R*-factors *R* are based on *F*, with *F* set to zero for negative *F*^2^. The observed criterion of *F*^2^ > σ(*F*^2^) is used only for calculating -*R*-factor-obs *etc*. and is not relevant to the choice of reflections for refinement. *R*-factors based on *F*^2^ are statistically about twice as large as those based on *F*, and *R*-factors based on ALL data will be even larger.

### Fractional atomic coordinates and isotropic or equivalent isotropic displacement parameters (Å^2^)

**Table:**

	*x*	*y*	*z*	*U*_iso_*/*U*_eq_	
Br1	0.4208 (2)	0.8732 (4)	0.60524 (11)	0.0799 (7)	
Br2	1.11644 (19)	0.3412 (4)	0.60918 (10)	0.0766 (7)	
O1	0.9808 (12)	0.057 (2)	0.7220 (5)	0.069 (4)	
C1	0.7790 (16)	0.247 (3)	0.6578 (8)	0.062 (2)	
C2	0.7029 (16)	0.359 (3)	0.7090 (8)	0.062 (2)	
C3	0.5983 (16)	0.544 (3)	0.6919 (8)	0.062 (2)	
C4	0.5687 (16)	0.618 (3)	0.6270 (7)	0.062 (2)	
C5	0.6461 (16)	0.517 (3)	0.5754 (8)	0.062 (2)	
C6	0.7444 (16)	0.331 (3)	0.5936 (8)	0.062 (2)	
C7	0.8485 (19)	0.172 (3)	0.5499 (7)	0.067 (6)	
C8	0.9613 (18)	0.074 (3)	0.5978 (7)	0.063 (6)	
C9	0.8931 (16)	0.040 (3)	0.6617 (7)	0.057 (5)	
H1	1.00390	−0.09700	0.73430	0.1030*	
H2	0.72330	0.30880	0.75290	0.0740*	
H3	0.54710	0.62080	0.72470	0.0740*	
H5	0.62990	0.57520	0.53190	0.0740*	
H7A	0.80100	0.02070	0.52700	0.0810*	
H7B	0.88630	0.29260	0.51750	0.0810*	
H8	0.99730	−0.10210	0.58330	0.0750*	
H9	0.84820	−0.14010	0.66060	0.0680*	

### Atomic displacement parameters (Å^2^)

**Table:**

	*U*^11^	*U*^22^	*U*^33^	*U*^12^	*U*^13^	*U*^23^
Br1	0.0591 (11)	0.0778 (13)	0.1011 (14)	0.0087 (10)	−0.0058 (10)	0.0030 (11)
Br2	0.0611 (11)	0.0755 (12)	0.0942 (13)	0.0033 (9)	0.0123 (9)	0.0108 (10)
O1	0.081 (8)	0.052 (6)	0.072 (7)	0.005 (6)	−0.002 (6)	0.004 (5)
C1	0.052 (4)	0.069 (4)	0.064 (4)	−0.007 (3)	0.001 (3)	−0.001 (3)
C2	0.052 (4)	0.069 (4)	0.064 (4)	−0.007 (3)	0.001 (3)	−0.001 (3)
C3	0.052 (4)	0.069 (4)	0.064 (4)	−0.007 (3)	0.001 (3)	−0.001 (3)
C4	0.052 (4)	0.069 (4)	0.064 (4)	−0.007 (3)	0.001 (3)	−0.001 (3)
C5	0.052 (4)	0.069 (4)	0.064 (4)	−0.007 (3)	0.001 (3)	−0.001 (3)
C6	0.052 (4)	0.069 (4)	0.064 (4)	−0.007 (3)	0.001 (3)	−0.001 (3)
C7	0.089 (12)	0.058 (9)	0.056 (8)	0.007 (9)	0.013 (8)	−0.005 (8)
C8	0.083 (12)	0.046 (8)	0.058 (9)	0.008 (8)	−0.005 (8)	0.004 (7)
C9	0.059 (9)	0.048 (8)	0.061 (9)	−0.003 (7)	−0.008 (8)	0.010 (7)

### Geometric parameters (Å, º)

**Table:**

Br1—C4	1.910 (15)	C6—C7	1.58 (2)
Br2—C8	1.974 (16)	C7—C8	1.47 (2)
O1—C9	1.430 (18)	C8—C9	1.50 (2)
O1—H1	0.8200	C2—H2	0.9300
C1—C6	1.38 (2)	C3—H3	0.9300
C1—C9	1.48 (2)	C5—H5	0.9300
C1—C2	1.42 (2)	C7—H7A	0.9700
C2—C3	1.37 (2)	C7—H7B	0.9700
C3—C4	1.37 (2)	C8—H8	0.9800
C4—C5	1.41 (2)	C9—H9	0.9800
C5—C6	1.34 (2)		
			
C9—O1—H1	109.00	O1—C9—C1	112.6 (12)
C2—C1—C6	118.3 (14)	C1—C2—H2	121.00
C6—C1—C9	112.2 (13)	C3—C2—H2	121.00
C2—C1—C9	129.5 (14)	C2—C3—H3	120.00
C1—C2—C3	118.1 (15)	C4—C3—H3	120.00
C2—C3—C4	120.7 (15)	C4—C5—H5	122.00
Br1—C4—C5	118.3 (11)	C6—C5—H5	122.00
C3—C4—C5	122.3 (14)	C6—C7—H7A	111.00
Br1—C4—C3	119.4 (11)	C6—C7—H7B	111.00
C4—C5—C6	115.3 (15)	C8—C7—H7A	111.00
C1—C6—C7	105.3 (12)	C8—C7—H7B	111.00
C5—C6—C7	129.4 (14)	H7A—C7—H7B	109.00
C1—C6—C5	125.2 (15)	Br2—C8—H8	110.00
C6—C7—C8	104.4 (12)	C7—C8—H8	110.00
Br2—C8—C9	109.9 (10)	C9—C8—H8	110.00
C7—C8—C9	105.3 (13)	O1—C9—H9	107.00
Br2—C8—C7	111.3 (10)	C1—C9—H9	107.00
O1—C9—C8	117.9 (13)	C8—C9—H9	107.00
C1—C9—C8	103.8 (12)		
			
C6—C1—C2—C3	0 (2)	Br1—C4—C5—C6	−177.5 (11)
C9—C1—C2—C3	−178.1 (15)	C3—C4—C5—C6	4 (2)
C2—C1—C6—C5	2 (2)	C4—C5—C6—C1	−4 (2)
C2—C1—C6—C7	179.4 (13)	C4—C5—C6—C7	179.1 (14)
C9—C1—C6—C5	−179.5 (15)	C1—C6—C7—C8	−16.7 (16)
C9—C1—C6—C7	−1.9 (17)	C5—C6—C7—C8	160.8 (16)
C2—C1—C9—O1	−33 (2)	C6—C7—C8—Br2	−90.7 (12)
C2—C1—C9—C8	−162.1 (15)	C6—C7—C8—C9	28.3 (15)
C6—C1—C9—O1	148.1 (13)	Br2—C8—C9—O1	−34.8 (16)
C6—C1—C9—C8	19.4 (17)	Br2—C8—C9—C1	90.6 (12)
C1—C2—C3—C4	0 (2)	C7—C8—C9—O1	−154.8 (12)
C2—C3—C4—Br1	179.3 (12)	C7—C8—C9—C1	−29.4 (15)
C2—C3—C4—C5	−2 (2)		

### Hydrogen-bond geometry (Å, º)

Cg2 is the centroid of the C1–C6 benzene ring.

**Table:**

*D*—H···*A*	*D*—H	H···*A*	*D*···*A*	*D*—H···*A*
O1—H1···O1^i^	0.82	1.91	2.713 (14)	165
C9—H9···*Cg*2^ii^	0.98	2.67	3.629 (16)	166

Symmetry codes: (i) −*x*+2, *y*−1/2, −*z*+3/2; (ii) *x*, *y*−1, *z*.
